# The transfer of titanium dioxide nanoparticles from the host plant to butterfly larvae through a food chain

**DOI:** 10.1038/srep23819

**Published:** 2016-03-31

**Authors:** Miyoko Kubo-Irie, Masaaki Yokoyama, Yusuke Shinkai, Rikio Niki, Ken Takeda, Masaru Irie

**Affiliations:** 1 Center for Environmental Health Science for the Next Generation, Research Institute for Science and Technology, Tokyo University of Science, 2641 Yamazaki, Noda-shi, Chiba 261-8502 Japan; 2 Biological Laboratory, The Open University of Japan, Wakaba, Mihama-ku, Chiba 261-8506, Japan; 3 Application Center-Tokyo, Research and Development Division, HORIBA, LTD., 2-6 Awajicho, Kanda, Chiyoda-Ku, Tokyo 101-0063, Japan; 4 Department of Computer Science, Waseda University, 3-4-1 Ohkubo, Shinjuku-ku, Tokyo 169-8555, Japan

## Abstract

This study aimed to examine the transfer of nanoparticles within a terrestrial food chain. Oviposited eggs of the swallowtail butterfly (*Atrophaneura alcinous*) were hatched on the leaves of the host plant (*Aristolochia debilis*), and the root stock and root hairs were submerged in a suspension of 10 μg/ml titanium dioxide nanoparticles (TiO_2_-NPs) in a 100 ml bottle. The presence of TiO_2_-NPs in the veins of the leaves was confirmed by X-ray analytical microscopy (X-ray AM). The hatched 1st instar larvae fed on the leaves to moult into 2nd instar larvae. Small agglomerates of TiO_2_-NPs less than 150 nm in diameter were identified in the vascular tissue of the exposed plant, the midgut and the excreta of the larvae by transmission electron microscopy. The image of Ti elemental mapping by X-ray AM was analysed with the quantitative spatial information mapping (QSIM) technique. The results demonstrated that TiO_2_-NPs were transferred from the plant to the larvae and they were disseminated throughout the environment via larval excreta.

As nanotechnology becomes more widespread, manufactured nanomaterials are latently discharged into the environment. The potential environmental risks to human health have been the subject of increasing attention^1^,^2^,^3^,^4^,^5^,^6^,^7^. Titanium dioxide nanoparticles (TiO_2_-NPs) have been considered of low harm to human health and are widely used in cosmetics and as food additives^8^. However, investigations into the environmental fate, the potential bioaccumulation and, in particular, the transfer of NPs throughout the food chain remain limited.

The food chain transfer of TiO_2_-NPs in an aquatic environment was reported via the oral uptake of zooplankton (*Daphnia magna*), which transferred TiO_2_-NPs to zebrafish (*Danio rerio*) through aqueous and dietary exposure in kinetic models. The ecological risk assessments and the potential for biomagnification were of concern^9^. Silver NPs (Ag-NPs) were shown to be transferred from a phytoplankton food source (*Ankistrodesmus falcatus*) to a zooplankton grazer (*D. magna*) by UV-Vis spectroscopy^10^. The bioaccumulation of carboxylated and biotinylated quantum dot NPs from water in ciliate protozoa (*Tetrahymena pyriformis*) to a planktonic rotifer (*Brachionus calyciflorus*) was observed to have occurred through dietary uptake by confocal laser scanning microscopy and light microscopy^11^. Furthermore, the transfer of polystyrene NPs from algae (*Scenedesmus* sp.) and zooplankton (*D. magna*) to a fish (*Carassius carassius*) through a food chain has been observed by fluorescence microscopy^12^.

In the terrestrial environment, food chain transfers generally occur from plants to animals. As a primary producer in the food chain, the roots of plants absorb nutrients and water with contaminants from the environment. Translocation and accumulation have been studied using analytical instruments to detect NPs in various plant tissues, including ZnO-NPs in ryegrass (*Lolium perenne*)^13^; C_70_-NPs in rice seeds (*Oryza sativa*)^14^; Cu-NPs in mung bean (*Phaseolus radiates*), wheat (*Triticum aestivum*)^15^ and maize seedlings (*Zea mays*)^16^; Au-NPs in rattlebush (*Sesbania drummondii*) seeds^17^ and tobacco (*Nicotiana xanthi*) seedlings^18^; Fe_3_O_4_-NPs in pumpkin plants (*Cucurbita maxima*)^19^; La_2_O_3_-NPs in cucumber (*Cucumis sativus*)^20^; Ag-NPs in thale cress (*Arabidopsis thaliana*) seeds (a flowering plant)^21^ and in *O. sativa* seeds^22^. NPs in these plant models have been found to cause leaf necrosis, inhibit seedling root elongation and affect root growth.

In terrestrial transfer and biomagnification, Au-NPs were first demonstrated in the host plant *Nicotiana tabacum* and the 2nd instar larval midgut of the tobacco hornworm (*Manduca sexta*) by inductively coupled plasma-mass spectrometry (ICP-MS) and synchrotron X-ray fluorescence maps (*μ*XRF)^23^.

Herein, we examine the transfer of TiO_2_-NPs through the trophic food chain from a host plant (*Aristolochia debilis*) to the larvae of the swallowtail butterfly (*Atrophaneura alcinous*) using different evaluation methods, including transmission electron microscopy (TEM) and the newly developed method of X-ray analytical microscopy (X-ray AM).

## Results

### Characterisation of TiO_2_-NPs

**Figure 1:**
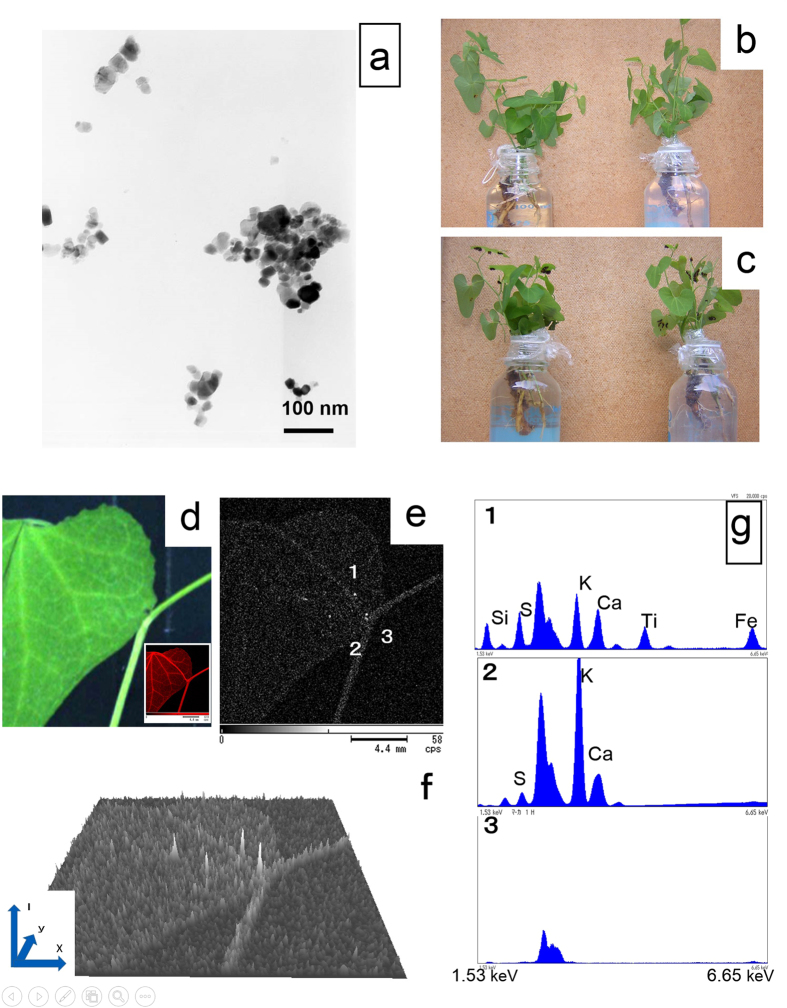
Scheme of experiments. (**a**) TEM image of the TiO_2_-NPs shows that agglomerates were composed of a small number of TiO_2_-NPs. Individual TiO_2_-NPs of 35 nm in primary diameter were spherical or rod-shaped and were held together by relatively weak forces. (**b**) Photographs of the plant exposure setup in the experiment. The root stock and root hairs of a plant with leaves containing oviposited eggs was submerged in the 100 ml bottle. The bottle contained either the TiO_2_-NP suspension (exposure plant, right-hand bottle) or distilled water (control plant, left-hand bottle). The 10 μg/ml TiO_2_-NPs in the bottle was slightly white, whereas the distilled water in the bottle was clear. (**c**) After 48 hours, the 1st instar larvae hatched and began to feed on the leaves. (**d**) Leaves with stems were examined by X-ray AM. The distribution of K element in the square was rich in the veins of the leaf and the stem. **(e)** The localized high intensity dots show the possibility of existence TiO_2_-NPs on the leaves. **(f)**. The QSIM image of Ti obtained with originally developed computer code QSIM-3D. The vertical axis shows the intensity (I) of the spectrum at the position (x,y). **(g)** The elemental spectrum (1 in Fig. 1e) showed the characteristic peak for Ti of 4.51 keV. The elemental spectrum with low intensity dots (2 in Fig. 1e) showed a negligible peak for Ti compared with the other elements including K and Ca. There was no characteristic peak in the X-ray AM holding stage (3 in Fig. 1e).

The TEM micrographs demonstrated the shapes and sizes of the NPs. Some TiO_2_ agglomerates were composed of a small number of NPs that were held together by relatively weak forces. Individual TiO_2_-NPs of 35 nm in primary diameter were spherical or rod shaped (Fig. 1a). The Z-average was 78.68 nm and the polydispersity index was 0.20; a narrow size distribution was indicated by the dynamic light scattering measurement.

### Exposure of plants to TiO_2_-NPs

**Figure 2:**
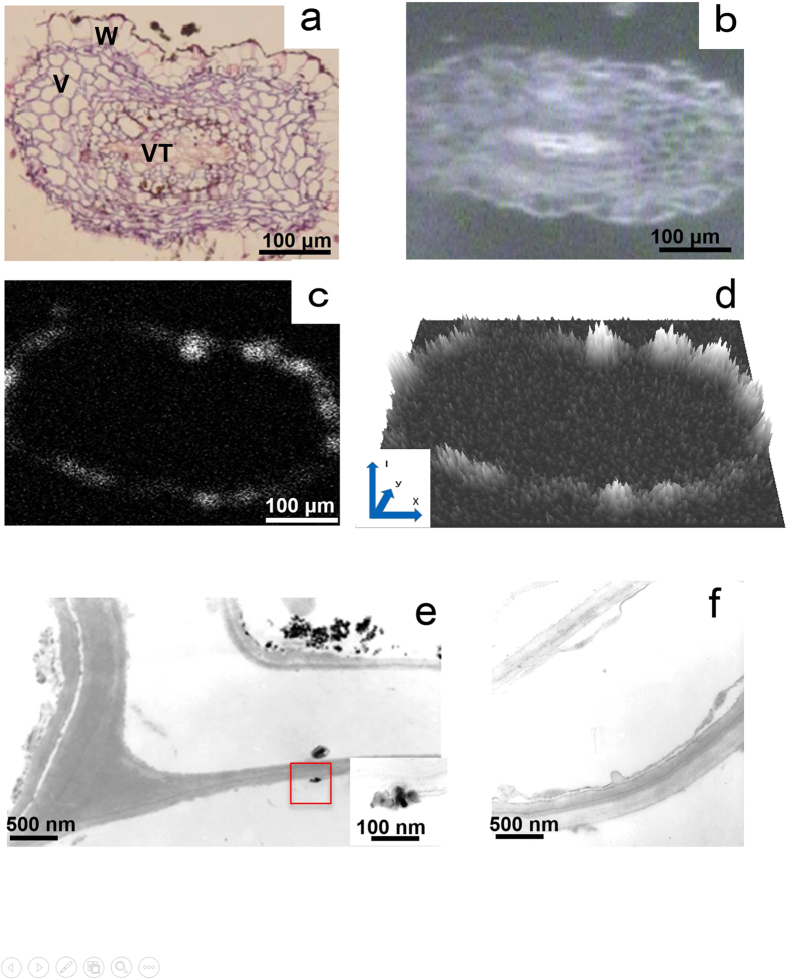
TiO_2_-NPs in the root hair. (**a**) A cross-section of an *A. debilis* root hair on a light microscopy image showing the vascular tissue (VT) in the centre and large vacuoles (V) in the periphery (HE staining). Some TiO_2_-NP aggregates were found on the surface of the thin cell wall (W). (**b**) X-ray AM image of the cross-section of a root hair. (**c**) Ti was detected on the surface of the cell wall in the same section as on the Ti mapping image. (**d**) The QSIM image taken from of Fig. 2c, showed strong spectral peak concentrated outside the cell wall. The vertical axis shows the intensity (I) of the spectrum at the position (x,y). (**e**) The TEM image reveals the presence of numerous aggregates on the surface of the cell wall and small agglomerates found near the cellulose layer of epidermal cells. Under high magnification (square in Fig. 2d), the agglomerate contains several TiO_2_-NPs. (**f**) TEM image of a control plant showing no agglomerates on the surface of the cell wall and inside the epidermal cells.

**Figure 3:**
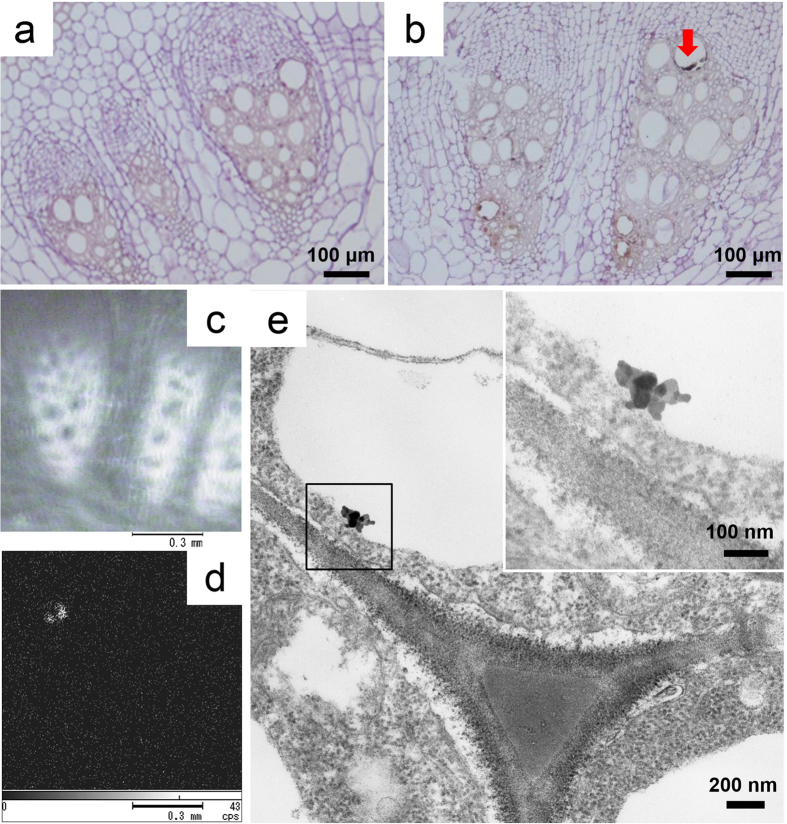
TiO_2_-NPs in the root vascular tissue. (**a**) The vascular tissue of the root stock from a control plant (HE staining) was observed by light microscopy. The lumen of the vascular tissue showed no agglomerates. (**b**) In the vascular tissue of the root stock from an exposed plant (HE staining), some agglomerates were observed by light microscopy (arrow). (**c**) The vascular tissue was examined by X-ray AM. (**d**) The uptake of TiO_2_-NPs was confirmed in this same section on the Ti mapping image. (**e**) TEM image shows TiO_2_-NPs in the vascular tissue and a small agglomerate composed of several TiO_2_-NPs under high magnification in the square (Fig. 3e).

The experiment was performed on plants with oviposited eggs. The liquid in the bottle without TiO_2_-NPs was clear, whereas that in the bottle with TiO_2_-NPs was slightly white (Fig. 1b).The colour remained consistent during exposure with no sedimentation in this condition, as described in the Materials and Methods. After 48 hours, the 1st instar larvae hatched and began to feed on the leaves of both the control and exposed plants (Fig. 1c). The absorbed water was approximately 35 ml with 10 μg/ml TiO_2_-NPs suspension for 7 days, and roughly calculated, 350 μg TiO_2_-NPs could be taken into the plant. The plants were not affected by exposure to TiO_2_-NPs. The leaves and stems were examined to confirm the uptake of TiO_2_-NPs in the veins by X-ray AM (Fig. 1d). The elemental mapping image (the square at the bottom right in Fig. 1d) of K was rich in the veins of the leaf and the stem, however, Ti image is scarce and recorded as high intensity dots (Fig. 1e). From Fig. 1e, we can obtain the quantitative spatial intensity mapping (QSIM) image of Ti (Fig. 1f) with originally developed computer code QSIM-3D. Four localized spectral peak each within the area of about 0.04 mm^2^ with the intensity. The elemental spectrum from the high intensity dot (1 in Fig. 1e) and the less bright area as control : (2 in Fig. 1e) and the X-ray AM holding stage (3 in Fig. 1e) was taken and displayed in the graph Fig. 1g, Figs 2, 3 consecutively. The characteristic peak for Ti of 4.51 keV was found clearly in Fig. 1g and not in control area.

### TiO_2_-NPs in the root hairs

The cross-section of an *A. debilis* root hair, which consisted of the vascular tissue in the centre and large vacuoles of epidermal cells in the periphery surrounded by a thin cell wall, was stained with haematoxylin and eosin (HE) in the exposed plant and observed by light microscopy (Fig. 2a). The cross-section was also examined by X-ray AM (Fig. 2b), in which numerous aggregates identified as Ti from the mapping image were found to be lining the cell wall (Fig. 2c). The QSIM image in Fig. 2d, taken from of Fig. 2c, showed strong spectral peak are concentrated outside the cell wall. In the TEM images, various numbers of TiO_2_-NP aggregates were attached to the surface of the thin cell wall (Fig. 2e), as indicated in the Ti mapping image (Fig. 2c). Small agglomerates were also found in the vacuoles of the epidermal cells near the cellulose layer. The high-magnification image in the square showed TiO_2_-NPs similar to those in the original TiO_2_-NP suspension (Fig. 1a). This image suggests that the agglomerates of TiO_2_-NPs smaller than 100 nm in diameter may cross the cellulose layer. No agglomerates were found on the surface of the thin cell wall and in the vacuoles of the epidermal cells in the control plant (Fig. 2f).

### TiO_2_-NPs in the root vascular tissue

Figure 3a shows the HE-stained vascular tissue of the root stock in a control plant. The lumen of the vascular tissue contains no agglomerates. In the exposed plant (Fig. 3b), however, the presence of some agglomerates in the vascular tissue was confirmed by X-ray AM, as shown on the X-ray image (Fig. 3c) and Ti mapping image of the vascular tissue (Fig. 3d). The TEM image shows the degeneration of cellular cytoplasm and a small agglomerate of TiO_2_-NPs. The high-magnification image in the square shows an agglomerate consisting of a few NPs (Fig. 3e).

### TiO_2_-NPs transfer to the larval midgut

**Figure 4:**
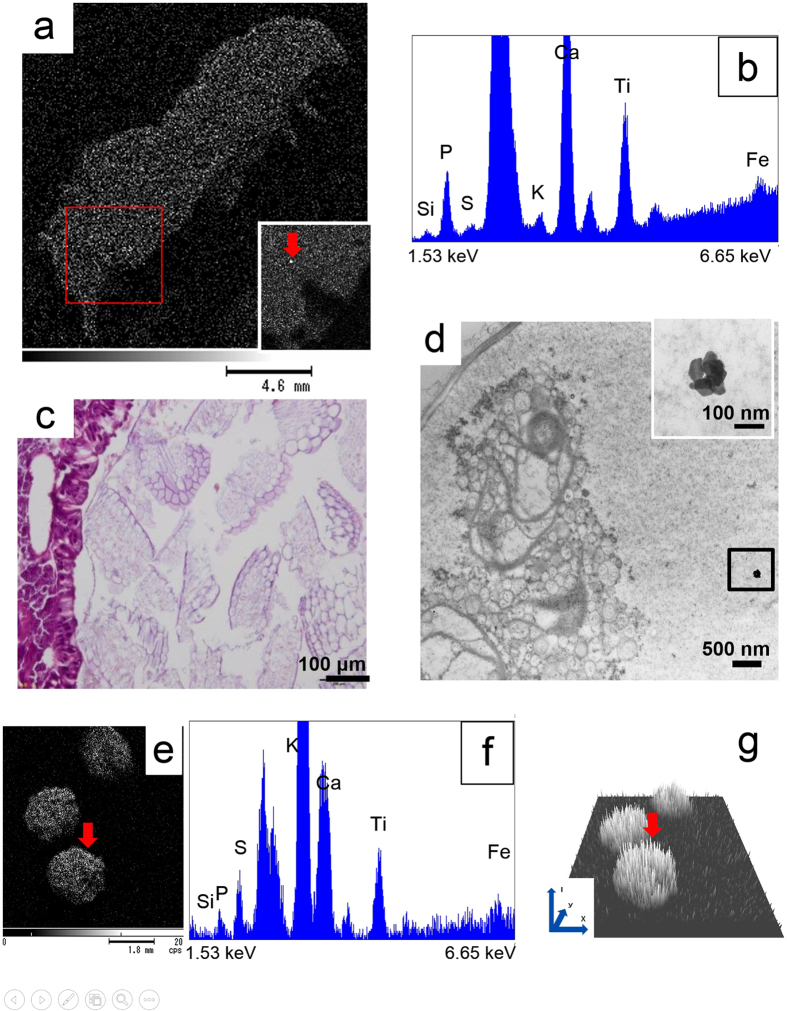
TiO_2_-NPs in the larval midgut and excreta. (**a**) A whole 2nd instar larvae without any treatment was examined by X-ray AM. A small agglomerate of TiO_2_-NPs in the square was indicated by Ti mapping (arrow). (**b**) Ti was indicated by a peak in the elemental spectrum at 4.51 keV. (**c**) Cross-section image of a 2nd instar larva (HE staining). Undigested leaves occupied the mid-gut. (**d**) In the TEM image of the mid-gut, a small agglomerate of TiO_2_-NPs in the square was found in the undigested leaf fragments as magnified. (**e**) Ti mapping image of excretal matter examined by X-ray AM. (**f**) Ti is observed in the excreta via the voltage peak in the elemental spectrum. (**g**) The QSIM image taken from Fig. 4e showed many high intensity spectral peaks and clearly distinguished the noise found on the holding stage. The vertical axis shows the spectral intensity (I) at the position (x,y).

The whole 2nd instar larva fed the exposed plant for one week was examined *in vivo* by X-ray AM under atmospheric pressure without any special treatment. In Fig. 4a, the head is at the top right and the anus is at the bottom left. A high intensity dot in the square indicates the localisation of TiO_2_-NPs around the anus, which was confirmed on the Ti elemental mapping image and on the elemental spectrum (Fig. 4b). Undigested leaf fragments were found to occupy the midgut in the HE-stained section of the larval midgut in the light microscopy image (Fig. 4c). A TEM image of the undigested leaf fragment in the square showed a small agglomerate of TiO_2_-NPs of <150 nm in diameter. This result suggested that the TiO_2_-NPs were transferred from the host plant to the larva via the trophic food chain. The larval excreta were also examined by X-ray AM. A high intensity dot of the larval excreta was shown to be Ti on the mapping image (arrow in Fig. 4e) and on the elemental spectrum (Fig. 4f). The QSIM image (Fig. 4g) taken from Fig. 4e showed many high intensity spectral peaks and clearly distinguished from the noise found on the holding stage.

## Discussion

The present work is the first indication obtained *in vivo* with X-ray AM of the transfer of NPs in the trophic food chain. Conventional energy-dispersive X-ray spectroscopy uses electron beams to irradiate the sample, which requires that the sample be placed in a vacuum. By contrast, X-ray AM applies X-ray flux configured by a specially designed “guide-tube” that can collimate down to 10 μm in diameter and can be applied to the sample at atmospheric pressure. This technique allows us to obtain the distribution of NPs in the “living” organism, as shown in Fig. 1d, and, particularly in the present study, a whole living larva (Fig. 4a) without any treatment.

We speculated that 350 μg of TiO_2_-NPs might be absorbed in the plant. However, aggregates remained on the surface of the root hair. From the QSIM image (Fig. 2d), the quantitative spatial intensity distribution of Ti were also demonstrated that the surface of the root hair was higher than the leaf stem. The aggregates might be too large to pass through the cell wall. The TEM image demonstrated only small agglomerates in the epidermal cells (the square in Fig. 2e) and in the vascular tissue (Fig. 3e). Therefore, much less than 350 μg of TiO_2_-NPs were absorbed *in vivo*. The Au-NPs biomagnification in the larva from the plant was indicated by ICP-MS and synchrotron X-ray fluorescence maps^23^. The TiO_2_-NPs transfer of a trophic food chain from the environment by the plant to the larva suggested to be small in the present study.

TiO_2_-NPs were not found in the midgut epithelium of the 2nd instar larvae. The limited bio-concentration of TiO_2_-NPs in the midgut epithelium may impede the detection of NPs. The effects of TiO_2_-NP-contaminated food on larval growth currently remain unknown, because of the short experimental period (only 7 days), during which time the oviposited eggs hatched and then the larvae fed on the leaves until they reached the 2nd instar larval stage. Different from previous studies^16^,^17^,^18^,^21^, the root hairs had already developed. In experiments conducted by Thuesombat *et al*. in which rice seeds were exposed to a range of concentrations of Ag-NPs (0.1 to 1000 mg/L), leaf deformation was not observed when the seeds were treated with Ag-NPs of 150 nm in diameter at concentrations of 10 or 100 mg/L during seed germination^22^. This finding showing that the low concentrations of Ag-NPs in rice seeds did not affect seed growth agreed with the results of our experiment.

Our primary focus in this work was a trophic food chain in which nanomaterials were transferred from a host plant to insect larvae. In a simulated experiment, 5th instar caterpillars of the tobacco hornworm (*M. sexta*) that fed on tomato leaf tissue whose surface was coated with 1 μg of Au-engineered nanomaterials (Au-ENM) were examined for growth, mortality and ingestion after 0, 1, 4 and 7 days. There was no difference between the control and treatment groups. Au-ENM accumulation in the gut was found to be low with ICP-MS and *μ*XRF analysis^24^. In our previous study, instead of ingestion via oral uptake, TiO_2_-NPs were subcutaneously injected into 5th instar larvae of the sweet potato hornworm (*Agrius convolvuli*). This examination did not disturb the pupation of the larvae or emergence of the pupae^25^.

In another study, the effects of Ag-NPs on the growth and feeding responses of two lepidopteran pests of the castor plant *(Ricinus communis*), the Asian armyworm (*Spodoptera litura*) and castor semilooper (*Achaea janata*) larvae, were investigated. Castor leaves treated with Ag-NPs were shown to accumulate in the larval guts by TEM observation, but the majority of Ag-NPs were eliminated through the faeces. The activities of superoxide dismutase, catalase and peroxidase were altered in the larval bodies as a result of the Ag-NP treatment^26^. TEM observation of the NPs in insect gut cells would help to provide better insight into their accumulation and localisation in cellular organelles, as confirmed in the present study. TiO_2_-NPs eliminated through the faeces might pose a potential environmental hazard. Our results revealed important eco-toxicological information regarding the dissemination of TiO_2_-NPs into the environment by the terrestrial food chain even though the quantity of NP agglomerates was small for each individual insect.

## Materials and Methods

### Characterisation of TiO_2_-NPs

A well-dispersed suspension of both the anatase and rutile forms of TiO_2_-NPs (80/20) purchased from Sigma-Aldrich Chem (product #700347, Switzerland) was used for this experiment. TiO_2_-NPs at 10 μg/ml dilution were dispersed in ultrapure water and ultrasonicated for 30 min. A drop of suspension was then placed on Formvar-coated copper grids and fully drained. The grids were allowed to air dry. The visualisation of the size and shape of the TiO_2_-NPs was achieved using a JEM 1200 EX II transmission electron microscope (JEOL Ltd., Japan). The size distribution of the TiO_2_-NPs included the intensity-weighted average diameter of the all-size population (Z-average). The polydispersity index was analysed by dynamic light scattering measurement using a Nano-ZS (Sysmex Co., Kobe, Japan).

### Host plant and the swallowtail larvae

The wild nettle (*A. debilis*) grows naturally on the campus of the Open University of Japan and in the private gardens around Urayasu, Chiba, Japan, from May to September. At the same time, *A. alcinous* butterflies appear, each of which lays several eggs on the backs of the nettle leaves. Ovipositing mothers play an important role in locating and recognising a suitable host plant for larval growth. They have a fifth-voltine life cycle during the long daytime period (16 hours of light/8 hours of darkness)^27^.

### Exposure

The root stock and root hairs of plants with leaves that had 5–10 oviposited eggs were submerged in a bottle filled with distilled water containing 10 μg/ml TiO_2_-NPs. The amount of evaporated water was replenished and the bottle was shaken gently by hand every day. Approximately 350 μg TiO_2_-NPs were in the water during the experiment. After 48 hours exposure, the 1st instar larvae hatched from eggs and fed on the leaves until they moulted into 2nd instar larvae. As a control, the root stock and root hairs of a plant with oviposited eggs and roots with similar characteristics were submerged in distilled water without TiO_2_-NPs under conditions that reflected outdoor temperatures (27–32 °C). Whole 2nd instar larvae of 12.5–13.8 mm in length were examined.

### X-ray analytical microscopy

After 7 days, the host plant and the whole 2nd instar larvae from exposed plants were examined for the presence of TiO_2_-NPs using an X-ray analytical microscope (XGT-5200, HORIBA, Japan) in atmospheric condition *in vivo*, without any special treatment. Ti was detected by examining elemental mapping images and by the presence of its characteristic 4.51 keV peak in the elemental spectrum.

### Tissue preparation for optical microscopy

The specimens of root hair, root stock, terrestrial stem, leaf and the 2nd instar larvae were fixed in 10% neutral buffered formalin. They were then dehydrated in an ethanol series and xylene, embedded in paraffin, cut into 5-μm sections and mounted on glass slides. Following paraffin removal and rehydration, the sections were stained with HE. All images were acquired with an attached Leica DFC 300FX digital camera (Leica Microsystems Digital Imaging, Cambridge, UK).

### Tissue preparation for TEM

The specimens of root hair, root stock, terrestrial stem, leaf and cross-sections of 2nd larval mid-gut were directly placed into in the primary fixative, 2.5% glutaraldehyde in 0.2 M sodium cacodylate buffer at a pH of 7.4, for 24 hours and post-fixed with 1% OsO_4_ for 1 hour. The samples were dehydrated through graded levels of ethanol and embedded in Quetol 812 (Nissin EM, Tokyo, Japan). Ultrathin sections of 85 nm were made with a Leica EM UC6rt ultra-microtome (Leica Mikrosystem GmbH, Vienna, Austria), stained with uranyl acetate and lead citrate, and then observed by TEM.

### QSIM-3D: computer aided quantitative mapping code

In order to obtain the quantitative spatial information mapping (QSIM) image from the X-ray AM output, we had developed the three dimensional computer code “QSIM-3D” , using C++ language, OpenCV 2.4.11 (INTEL) as well as GLUT (OpenGL) utility under Visual Studio 2013 (Microsoft) environment. QSIM image was processed from the elemental mapping image (EMI). The EMI image is just like a map painted in two dimensional plane. On the other hand QSIM-3D is three dimensional map which we can see the height of the image if we tilt the map. For example, in Fig. 2c, EMI, we can see the local intensity spots. On the other hand in Fig. 2d we can see the intensity distribution in vertical direction and looks like mountain ranges. The vertical axis shows the spectral intensity (I) at the position (x,y). In this three dimensional mapping, we added colour information showing the intensity as well. The maximum intensity point is indicates as “white” and the darkest part of EMI (Black) is converted to “gray” in order to be distinguished easily.

## Additional Information

**How to cite this article**: Kubo-Irie, M. *et al*. The transfer of titanium dioxide nanoparticles from the host plant to butterfly larvae through a food chain. *Sci. Rep*. **6**, 23819; doi: 10.1038/srep23819 (2016).
